# Methyl 3-*O*-α-l-fucopyranosyl α-d-gal­acto­pyran­oside: a synchrotron study

**DOI:** 10.1107/S1600536812002279

**Published:** 2012-01-31

**Authors:** Lars Eriksson, Göran Widmalm

**Affiliations:** a Department of Material and Environmental Chemistry, Arrhenius Laboratory, Stockholm University, S-106 91 Stockholm, Sweden; b Department of Organic Chemistry, Arrhenius Laboratory, Stockholm University, S-106 91 Stockholm, Sweden

## Abstract

The title compound, C_13_H_24_O_10_ is the methyl glycoside of a structural element α-l-Fucp-(1→ 3)-α-d-Galp making up two thirds of the repeating unit in the capsular polysaccharide of Klebsiella K63. The conformation of the title compound is described by the glycosidic torsion angles ϕ_H_ = 55 (1)° and ψ_H_ = −24 (1)°. The hy­droxy­methyl group in the galactose residue is present in the *gauche*–*trans* conformation. In the crystal, O—H⋯O hydrogen bonds connect the disaccharide units into chains along the *a*-axis direction and further hydrogen bonds cross-link the chains.

## Related literature

The capsular polysaccharide (CPS) of Klebsiella K63 contains a repeating unit consisting of → 3)-α-d-GalpA–(1 → 3)-α-l-Fucp-(1 → 3)-α-d-Galp-(1 →, see: Joseleau & Marais (1979 ▶). For an investigation of the CPS S-156 from Klebsiella pneumoniae ATCC 316 46, see: Johansson *et al.* (1994 ▶) and of the CPS from Klebsiella pneumoniae I-1507, see: Guetta *et al.* (2003 ▶). For a fiber X-ray diffraction study of the Klebsiella K63 CPS, see: Elloway *et al.* (1980 ▶). For the synthesis, see: Baumann *et al.* (1988 ▶).
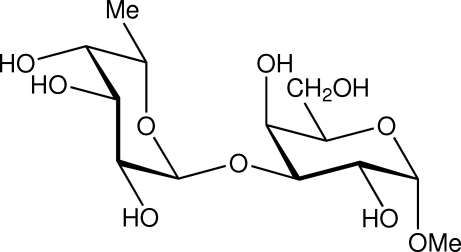


## Experimental

### 

#### Crystal data

C_13_H_24_O_10_
*M* *_r_* = 340.32
Orthorhombic, 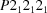

*a* = 4.78478 (11) Å
*b* = 15.7859 (5) Å
*c* = 19.4401 (5) Å
*V* = 1468.36 (7) Å^3^
*Z* = 4
Synchrotron radiation
λ = 0.907 Å
μ = 0.13 mm^−1^
*T* = 100 K
0.03 × 0.01 × 0.01 mm

#### Data collection

Marresearch MARCCD 165 diffractometer
7469 measured reflections
1162 independent reflections
975 reflections with *I* > 2σ(*I*)
*R*_int_ = 0.117
θ_max_ = 30.1°

#### Refinement

*R*[*F* ^2^ > 2σ(*F* ^2^)] = 0.060
*wR*(*F* ^2^) = 0.177
*S* = 1.09
1162 reflections
216 parameters
H-atom parameters constrained
Δρ_max_ = 0.30 e Å^−3^
Δρ_min_ = −0.27 e Å^−3^

### 

Data collection: *MARCCD* (Marresearch, 2010 ▶); cell refinement: *CrysAlis RED* (Oxford Diffraction, 2008 ▶); data reduction: *CrysAlis RED*; program(s) used to solve structure: *SHELXS97* (Sheldrick, 2008 ▶); program(s) used to refine structure: *SHELXL97* (Sheldrick, 2008 ▶); molecular graphics: *DIAMOND* (Brandenburg, 1999 ▶); software used to prepare material for publication: *PLATON* (Spek, 2009 ▶).

## Supplementary Material

Crystal structure: contains datablock(s) global, I. DOI: 10.1107/S1600536812002279/hb6569sup1.cif

Structure factors: contains datablock(s) I. DOI: 10.1107/S1600536812002279/hb6569Isup2.hkl

Additional supplementary materials:  crystallographic information; 3D view; checkCIF report

## Figures and Tables

**Table 1:** Hydrogen-bond geometry (Å, °)

*D*—H⋯*A*	*D*—H	H⋯*A*	*D*⋯*A*	*D*—H⋯*A*
O3f—H3f1⋯O6g^i^	0.84	1.93	2.772 (7)	177
O4f—H4f1⋯O3f^ii^	0.84	2.04	2.880 (7)	175
O2g—H2g1⋯O2g^iii^	0.84	1.92	2.680 (7)	149
O4g—H4g1⋯O5f^iv^	0.84	2.08	2.827 (6)	149
O6g—H6g⋯O3f^v^	0.84	2.02	2.822 (7)	158

Symmetry codes: (i) 
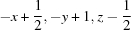
; (ii) 
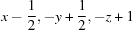
; (iii) 
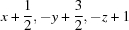
; (iv) 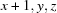; (v) 
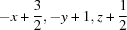
.

**Fig. 1.**
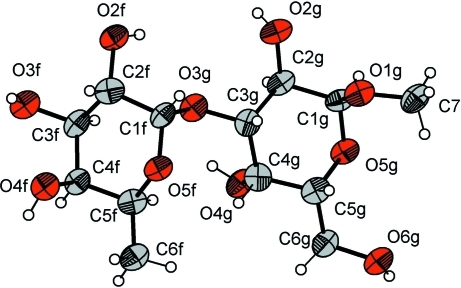
Molecular structure of I showing 50% probability displacement ellipsoids. The atom-label suffixes refer to the fucose(f) and galactose (g) residues. H atoms are shown as spheres of arbitrary radius.

**Fig. 2.**
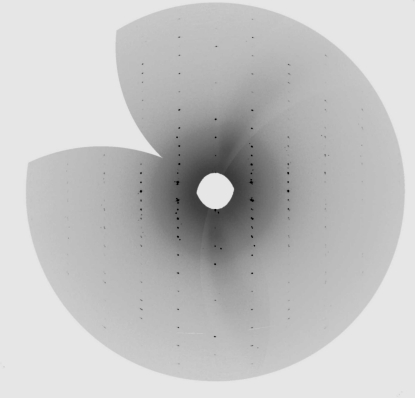
Reconstructed view of the H0L plane of reciprocal space

## supplementary crystallographic information

### Comment

Analysis of carbohydrate structure forms the basis of further studies related to interaction with other molecules and their function in different environments. Presently, the structural studies are often divided into the determination of the primary structure, *i.e.*, sugar residues and substituents including their absolute configuration, ring form, anomeric configuration and sequential arrangement of the constituent components. Subsequently, in the second part the three-dimensional structure is determined, often by NMR spectroscopy but also with X-ray diffraction (XRD) techniques when crystals of suitable quality and sufficient size are available.

Polysaccharides are often built of repeating units of oligosaccharides having two to seven sugar residues in their repeats. To understand the physicochemical properties and immunological specificity of the polymers it is essential to obtain information on their structures, both the primary and the three-dimensional structures.

The capsular polysaccharide (CPS) of Klebsiella K63 contains a repeating unit consisting of → 3)-α-D-GalpA– (1 → 3)-α-L-Fucp-(1 → 3)-α-D-Galp-(1 → (Joseleau *et al.*, 1979). More recently the CPS S-156 from Klebsiella pneumoniae ATCC 316 46 (Johansson *et al.*, 1994) and the CPS from Klebsiella pneumoniae I-1507 (Guetta *et al.*, 2003) were investigated. Their backbone structures were identical to that of the CPS from Klebsiella K63, *i.e.*, trisaccharide repeating units, except for stoichiometric *O*-acetylation at O4 of the galacturonic acid.

The physicochemical effects of *O*-deacetylation were investigated for 'Fucogel', *i.e.*, the CPS from strain I-1507 and revealed that the presence of the *O*-acetyl groups decreases the local stiffness of the polymer and lowers the rigidity of the polysaccharide as well as shortens the persistence length. The structural element α-L-Fucp-(1→3)-α-D-Galp makes up two thirds of the repeating unit in these polysaccharides and the title compound is the methyl glycoside thereof.

The torsion angles φ_H_, ψ_H_, and ω describe the major degrees of freedom in an oligosaccharide and for the title compound (I) the two former are present at the glycosidic α-(1 → 3)-linkage. In addition, for the galactose residue the φ_H_ torsion angle is also of interest. The ω torsion angle refers to the conformation of the hydroxymethyl group in the galactose residue. Both of the φ_H_ torsion angles in the structure are described by the *exo*-anomeric conformation with φ_H_ = 55 (1)° for the fucose residue and φ_H_ = -53 (1)° for the galactose residue (Fig. 1). The ψ_H_ torsion angle may in solution populate more that one conformational state (see below); for title compound (I) ψ_H_ = -24 (1)°. The conformation of the hydroxymethyl group is described by one of the three rotamers, *gauche*-*trans*, *gauche*-*gauche*, or *trans*-*gauche* with respect to the conformation of C6–O6 to C5–O5 and to C5–C4, respectively. In the present case the galactose residue has the gt conformation with ω = 70 (1)°, shifted away slightly from an ideal *gauche* conformation.

The Cremer-Pople parameters for the title compound are Q=0.525 (7) Å, θ=176.4 (8)° and φ=142 (10)° for the ring O5f → C5f and Q=0.556 (7) Å, θ=1.8 (7)° and φ=288 (14)° for the ring O5g → C5g; thus the conformation of both rings can be described as C-forms.

In the study of Fucogel the conformational space of the constituent disaccharides were investigated by molecular mechanics and Ramachandran maps. Two low energy regions were identified from the adiabatic map of α-L-Fucp-(1→ 3)-α-D-Galp with essentially equal potential energy at their minima being (i) φO5 = 279.6° and ψC4 = 140.4° and (ii) φO5 = 260.2° and ψC4 = 70.2°, in which the former torsion angle is defined by O5f—C1f—O3g—C3g and the latter by C1g—O3g—C3g—C4g. Interresidue hydrogen bonding was not present for these two conformations although it was identified for a significantly higher-energy conformation.

The conformation of the title compound I and the corresponding glycosidic torsion angles in the polysaccharide are indeed quite similar. The resemblance of the crystal structure and the two low-energy minima of the adiabatic map suggests that torsion angle information from XRD data may be suitable as starting points for molecular modeling of oligo- and polysaccharides.

Interestingly, a fiber X-ray diffraction study of the Klebsiella K63 CPS shows that it forms an extended 2-fold helix (Elloway *et al.*, 1980).

### Experimental

The synthesis of (I) was described by Baumann *et al.* (1988) in which the fucose and galactose residues have the *L* and D absolute configurations, respectively. The compound was crystallized by slow evaporation of a mixture of water and ethanol (1:1) at ambient temperature.

### Refinement

The hydrogen atoms were refined in riding mode with *U*_iso_(H) = 1.5*U*_eq_(*X*), where *X* = C or O. The coverage at 0.8 Å resolution = 0.738 but already at 0.9 Å resolution the coverage has increased to 0.922 and at 1.0 Å resolution the coverage ~0.995. The refinement with reflection data up to 1.0 Å resolution converged at R1 = 0.0466. It should be noted that the reflection data diminishes at high resolution as shown in Fig 2; thus the low coverage to 0.8 or 0.9 Å is of minor importance.

### Crystal data

**Table:**

C_13_H_24_O_10_	*Z* = 4
*M**_r_* = 340.32	*F*(000) = 728
Orthorhombic, *P*2_1_2_1_2_1_	*D*_x_ = 1.539 Mg m^−^^3^
Hall symbol: P 2ac 2ab	Synchrotron radiation, λ = 0.907 Å
*a* = 4.78478 (11) Å	µ = 0.13 mm^−^^1^
*b* = 15.7859 (5) Å	*T* = 100 K
*c* = 19.4401 (5) Å	Prism, colorless
*V* = 1468.36 (7) Å^3^	0.03 × 0.01 × 0.01 mm

### Data collection

**Table:**

Marresearch MARCCD 165 diffractometer	975 reflections with *I* > 2σ(*I*)
Radiation source: I911, Maxlab	*R*_int_ = 0.117
Si(111)	θ_max_ = 30.1°, θ_min_ = 3.1°
Detector resolution: 0.0806 pixels mm^-1^	*h* = −5→5
φ scans	*k* = −17→17
7469 measured reflections	*l* = −20→20
1162 independent reflections	

### Refinement

**Table:**

Refinement on *F*^2^	Primary atom site location: structure-invariant direct methods
Least-squares matrix: full	Secondary atom site location: difference Fourier map
*R*[*F*^2^ > 2σ(*F*^2^)] = 0.060	Hydrogen site location: inferred from neighbouring sites
*wR*(*F*^2^) = 0.177	H-atom parameters constrained
*S* = 1.09	*w* = 1/[σ^2^(*F*_o_^2^) + (0.1211*P*)^2^ + 0.7072*P*] where *P* = (*F*_o_^2^ + 2*F*_c_^2^)/3
1162 reflections	(Δ/σ)_max_ < 0.001
216 parameters	Δρ_max_ = 0.30 e Å^−^^3^
0 restraints	Δρ_min_ = −0.27 e Å^−^^3^

### Special details

**Table:**

Geometry. All e.s.d.'s (except the e.s.d. in the dihedral angle between two l.s. planes) are estimated using the full covariance matrix. The cell e.s.d.'s are taken into account individually in the estimation of e.s.d.'s in distances, angles and torsion angles; correlations between e.s.d.'s in cell parameters are only used when they are defined by crystal symmetry. An approximate (isotropic) treatment of cell e.s.d.'s is used for estimating e.s.d.'s involving l.s. planes.
Refinement. Refinement of *F*^2^ against ALL reflections. The weighted *R*-factor *wR* and goodness of fit *S* are based on *F*^2^, conventional *R*-factors *R* are based on *F*, with *F* set to zero for negative *F*^2^. The threshold expression of *F*^2^ > σ(*F*^2^) is used only for calculating *R*-factors(gt) *etc*. and is not relevant to the choice of reflections for refinement. *R*-factors based on *F*^2^ are statistically about twice as large as those based on *F*, and *R*- factors based on ALL data will be even larger.

### Fractional atomic coordinates and isotropic or equivalent isotropic displacement parameters (Å^2^)

**Table:**

	*x*	*y*	*z*	*U*_iso_*/*U*_eq_	
C1F	0.2242 (15)	0.5346 (4)	0.5260 (3)	0.0359 (16)	
H1F	0.0917	0.5802	0.5114	0.054*	
C2F	0.3048 (14)	0.4827 (5)	0.4636 (3)	0.0409 (17)	
H2F	0.1270	0.4633	0.4416	0.061*	
C3F	0.4713 (14)	0.4025 (4)	0.4827 (3)	0.0394 (17)	
H3F	0.6577	0.4217	0.4999	0.059*	
C4F	0.3298 (15)	0.3548 (5)	0.5419 (3)	0.0382 (17)	
H4F	0.4571	0.3089	0.5585	0.057*	
C5F	0.2588 (15)	0.4126 (4)	0.6012 (3)	0.0403 (17)	
H5F	0.4368	0.4347	0.6214	0.060*	
O5F	0.0941 (9)	0.4845 (3)	0.5765 (2)	0.0393 (12)	
C6F	0.0937 (17)	0.3710 (5)	0.6571 (3)	0.0465 (19)	
H6F1	0.0533	0.4124	0.6933	0.070*	
H6F2	0.2017	0.3240	0.6764	0.070*	
H6F3	−0.0822	0.3494	0.6381	0.070*	
O2F	0.4489 (10)	0.5325 (3)	0.4138 (2)	0.0430 (13)	
H2F1	0.5624	0.5648	0.4338	0.065*	
O3F	0.5214 (11)	0.3485 (3)	0.4262 (2)	0.0458 (13)	
H3F1	0.3725	0.3417	0.4040	0.069*	
O4F	0.0806 (9)	0.3171 (3)	0.5133 (3)	0.0430 (13)	
H4F1	0.0532	0.2697	0.5316	0.065*	
C1G	0.6262 (16)	0.7690 (5)	0.6550 (3)	0.0400 (17)	
H1G	0.7796	0.8113	0.6487	0.060*	
C2G	0.6339 (14)	0.7069 (4)	0.5935 (3)	0.0371 (17)	
H2G	0.8264	0.6823	0.5909	0.056*	
C3G	0.4279 (14)	0.6336 (4)	0.6047 (3)	0.0362 (16)	
H3G	0.2321	0.6554	0.6017	0.054*	
C4G	0.4759 (15)	0.5952 (5)	0.6746 (4)	0.0406 (18)	
H4G	0.3289	0.5514	0.6831	0.061*	
C5G	0.4618 (15)	0.6608 (4)	0.7312 (4)	0.0420 (18)	
H5G	0.2701	0.6860	0.7328	0.063*	
C6G	0.5295 (18)	0.6222 (5)	0.7996 (3)	0.0463 (19)	
H6G1	0.4176	0.5700	0.8059	0.069*	
H6G2	0.7295	0.6062	0.8006	0.069*	
O1G	0.3648 (10)	0.8128 (3)	0.6509 (2)	0.0434 (13)	
O2G	0.5808 (10)	0.7497 (3)	0.5311 (2)	0.0436 (13)	
H2G1	0.7044	0.7370	0.5022	0.065*	
O3G	0.4759 (10)	0.5731 (3)	0.5507 (2)	0.0412 (12)	
O4G	0.7492 (10)	0.5552 (3)	0.6797 (2)	0.0444 (13)	
H4G1	0.7904	0.5329	0.6418	0.067*	
O5G	0.6680 (10)	0.7279 (3)	0.7175 (2)	0.0418 (13)	
O6G	0.4725 (10)	0.6796 (3)	0.8553 (2)	0.0446 (13)	
H6G	0.5970	0.6748	0.8855	0.067*	
C7	0.3647 (18)	0.8825 (5)	0.6975 (3)	0.049 (2)	
H7A	0.3842	0.8615	0.7447	0.073*	
H7B	0.1885	0.9137	0.6931	0.073*	
H7C	0.5213	0.9202	0.6867	0.073*	

### Atomic displacement parameters (Å^2^)

**Table:**

	*U*^11^	*U*^22^	*U*^33^	*U*^12^	*U*^13^	*U*^23^
C1F	0.026 (3)	0.053 (4)	0.029 (3)	0.002 (3)	−0.004 (3)	−0.006 (3)
C2F	0.021 (3)	0.061 (4)	0.041 (4)	−0.004 (3)	−0.012 (3)	0.005 (4)
C3F	0.025 (3)	0.058 (4)	0.035 (4)	−0.001 (4)	−0.003 (3)	−0.007 (3)
C4F	0.028 (4)	0.051 (4)	0.035 (4)	−0.004 (3)	−0.007 (3)	−0.005 (3)
C5F	0.024 (3)	0.054 (4)	0.044 (4)	0.005 (4)	−0.003 (3)	0.006 (3)
O5F	0.023 (2)	0.057 (3)	0.038 (3)	0.000 (2)	0.002 (2)	0.002 (2)
C6F	0.038 (4)	0.062 (5)	0.040 (4)	0.007 (4)	0.001 (3)	0.000 (4)
O2F	0.030 (3)	0.061 (3)	0.038 (2)	−0.004 (2)	0.004 (2)	0.000 (2)
O3F	0.026 (3)	0.067 (3)	0.044 (3)	0.000 (3)	−0.002 (2)	−0.001 (2)
O4F	0.023 (3)	0.058 (3)	0.048 (3)	−0.005 (2)	0.000 (2)	−0.001 (2)
C1G	0.036 (4)	0.051 (4)	0.032 (4)	0.005 (4)	0.001 (3)	0.007 (3)
C2G	0.020 (3)	0.059 (4)	0.032 (3)	0.006 (3)	0.001 (3)	0.000 (3)
C3G	0.020 (3)	0.049 (4)	0.040 (4)	0.001 (3)	−0.012 (3)	−0.003 (3)
C4G	0.021 (3)	0.051 (4)	0.050 (4)	0.005 (3)	−0.007 (3)	0.000 (4)
C5G	0.025 (4)	0.059 (5)	0.043 (4)	0.002 (4)	−0.002 (3)	0.002 (3)
C6G	0.047 (5)	0.055 (4)	0.037 (4)	0.006 (4)	−0.002 (4)	−0.004 (4)
O1G	0.029 (3)	0.059 (3)	0.042 (3)	0.006 (3)	−0.006 (2)	0.006 (2)
O2G	0.023 (3)	0.071 (3)	0.038 (2)	0.008 (3)	0.002 (2)	0.008 (3)
O3G	0.025 (3)	0.058 (3)	0.041 (3)	0.000 (2)	−0.009 (2)	−0.002 (2)
O4G	0.031 (3)	0.063 (3)	0.040 (3)	0.010 (3)	0.003 (2)	−0.006 (2)
O5G	0.028 (3)	0.061 (3)	0.037 (3)	−0.005 (2)	−0.002 (2)	0.000 (2)
O6G	0.029 (3)	0.069 (3)	0.036 (3)	−0.003 (3)	−0.002 (2)	−0.004 (3)
C7	0.042 (4)	0.068 (5)	0.036 (4)	0.010 (4)	−0.008 (3)	−0.004 (4)

### Geometric parameters (Å, °)

**Table:**

C1F—O5F	1.406 (8)	C1G—C2G	1.546 (9)
C1F—O3G	1.432 (8)	C1G—H1G	1.0000
C1F—C2F	1.515 (9)	C2G—O2G	1.414 (8)
C1F—H1F	1.0000	C2G—C3G	1.535 (9)
C2F—O2F	1.425 (8)	C2G—H2G	1.0000
C2F—C3F	1.541 (10)	C3G—O3G	1.438 (8)
C2F—H2F	1.0000	C3G—C4G	1.505 (9)
C3F—O3F	1.411 (8)	C3G—H3G	1.0000
C3F—C4F	1.532 (10)	C4G—O4G	1.456 (9)
C3F—H3F	1.0000	C4G—C5G	1.513 (10)
C4F—O4F	1.444 (8)	C4G—H4G	1.0000
C4F—C5F	1.509 (9)	C5G—O5G	1.471 (8)
C4F—H4F	1.0000	C5G—C6G	1.498 (10)
C5F—O5F	1.462 (8)	C5G—H5G	1.0000
C5F—C6F	1.495 (10)	C6G—O6G	1.437 (8)
C5F—H5F	1.0000	C6G—H6G1	0.9900
C6F—H6F1	0.9800	C6G—H6G2	0.9900
C6F—H6F2	0.9800	O1G—C7	1.424 (8)
C6F—H6F3	0.9800	O2G—H2G1	0.8400
O2F—H2F1	0.8400	O4G—H4G1	0.8400
O3F—H3F1	0.8400	O6G—H6G	0.8400
O4F—H4F1	0.8400	C7—H7A	0.9800
C1G—O5G	1.392 (8)	C7—H7B	0.9800
C1G—O1G	1.431 (9)	C7—H7C	0.9800
			
O5F—C1F—O3G	112.2 (5)	O1G—C1G—H1G	108.2
O5F—C1F—C2F	111.6 (5)	C2G—C1G—H1G	108.2
O3G—C1F—C2F	106.5 (5)	O2G—C2G—C3G	111.5 (5)
O5F—C1F—H1F	108.8	O2G—C2G—C1G	110.9 (5)
O3G—C1F—H1F	108.8	C3G—C2G—C1G	110.7 (5)
C2F—C1F—H1F	108.8	O2G—C2G—H2G	107.9
O2F—C2F—C1F	111.7 (6)	C3G—C2G—H2G	107.9
O2F—C2F—C3F	111.5 (5)	C1G—C2G—H2G	107.9
C1F—C2F—C3F	112.5 (5)	O3G—C3G—C4G	111.5 (5)
O2F—C2F—H2F	106.9	O3G—C3G—C2G	107.1 (5)
C1F—C2F—H2F	106.9	C4G—C3G—C2G	109.5 (5)
C3F—C2F—H2F	106.9	O3G—C3G—H3G	109.6
O3F—C3F—C4F	111.3 (6)	C4G—C3G—H3G	109.6
O3F—C3F—C2F	113.4 (5)	C2G—C3G—H3G	109.6
C4F—C3F—C2F	110.9 (6)	O4G—C4G—C3G	111.9 (6)
O3F—C3F—H3F	107.0	O4G—C4G—C5G	106.7 (6)
C4F—C3F—H3F	107.0	C3G—C4G—C5G	112.0 (6)
C2F—C3F—H3F	107.0	O4G—C4G—H4G	108.7
O4F—C4F—C5F	110.9 (6)	C3G—C4G—H4G	108.7
O4F—C4F—C3F	106.1 (5)	C5G—C4G—H4G	108.7
C5F—C4F—C3F	112.1 (6)	O5G—C5G—C6G	108.0 (6)
O4F—C4F—H4F	109.2	O5G—C5G—C4G	109.2 (6)
C5F—C4F—H4F	109.2	C6G—C5G—C4G	111.0 (6)
C3F—C4F—H4F	109.2	O5G—C5G—H5G	109.5
O5F—C5F—C6F	107.1 (6)	C6G—C5G—H5G	109.5
O5F—C5F—C4F	109.8 (5)	C4G—C5G—H5G	109.5
C6F—C5F—C4F	114.1 (6)	O6G—C6G—C5G	111.7 (6)
O5F—C5F—H5F	108.5	O6G—C6G—H6G1	109.3
C6F—C5F—H5F	108.5	C5G—C6G—H6G1	109.3
C4F—C5F—H5F	108.5	O6G—C6G—H6G2	109.3
C1F—O5F—C5F	115.3 (5)	C5G—C6G—H6G2	109.3
C5F—C6F—H6F1	109.5	H6G1—C6G—H6G2	107.9
C5F—C6F—H6F2	109.5	C7—O1G—C1G	109.8 (5)
H6F1—C6F—H6F2	109.5	C2G—O2G—H2G1	109.5
C5F—C6F—H6F3	109.5	C1F—O3G—C3G	113.1 (5)
H6F1—C6F—H6F3	109.5	C4G—O4G—H4G1	109.5
H6F2—C6F—H6F3	109.5	C1G—O5G—C5G	113.4 (5)
C2F—O2F—H2F1	109.5	C6G—O6G—H6G	109.5
C3F—O3F—H3F1	109.5	O1G—C7—H7A	109.5
C4F—O4F—H4F1	109.5	O1G—C7—H7B	109.5
O5G—C1G—O1G	113.5 (5)	H7A—C7—H7B	109.5
O5G—C1G—C2G	112.0 (5)	O1G—C7—H7C	109.5
O1G—C1G—C2G	106.6 (5)	H7A—C7—H7C	109.5
O5G—C1G—H1G	108.2	H7B—C7—H7C	109.5
			
O5F—C1F—C2F—O2F	−177.1 (5)	O2G—C2G—C3G—O3G	64.1 (6)
O3G—C1F—C2F—O2F	−54.4 (7)	C1G—C2G—C3G—O3G	−172.1 (5)
O5F—C1F—C2F—C3F	−50.8 (7)	O2G—C2G—C3G—C4G	−174.9 (5)
O3G—C1F—C2F—C3F	71.9 (7)	C1G—C2G—C3G—C4G	−51.0 (7)
O2F—C2F—C3F—O3F	−60.3 (8)	O3G—C3G—C4G—O4G	53.0 (7)
C1F—C2F—C3F—O3F	173.3 (6)	C2G—C3G—C4G—O4G	−65.3 (7)
O2F—C2F—C3F—C4F	173.7 (5)	O3G—C3G—C4G—C5G	172.8 (6)
C1F—C2F—C3F—C4F	47.3 (7)	C2G—C3G—C4G—C5G	54.4 (7)
O3F—C3F—C4F—O4F	−55.3 (7)	O4G—C4G—C5G—O5G	65.6 (6)
C2F—C3F—C4F—O4F	71.8 (6)	C3G—C4G—C5G—O5G	−57.1 (7)
O3F—C3F—C4F—C5F	−176.6 (6)	O4G—C4G—C5G—C6G	−53.3 (8)
C2F—C3F—C4F—C5F	−49.5 (7)	C3G—C4G—C5G—C6G	−176.1 (6)
O4F—C4F—C5F—O5F	−64.9 (7)	O5G—C5G—C6G—O6G	69.9 (7)
C3F—C4F—C5F—O5F	53.6 (7)	C4G—C5G—C6G—O6G	−170.4 (6)
O4F—C4F—C5F—C6F	55.4 (8)	O5G—C1G—O1G—C7	67.5 (7)
C3F—C4F—C5F—C6F	173.9 (6)	C2G—C1G—O1G—C7	−168.6 (5)
O3G—C1F—O5F—C5F	−61.8 (6)	O5F—C1F—O3G—C3G	−65.4 (7)
C2F—C1F—O5F—C5F	57.6 (7)	C2F—C1F—O3G—C3G	172.3 (5)
C6F—C5F—O5F—C1F	176.5 (5)	C4G—C3G—O3G—C1F	97.7 (6)
C4F—C5F—O5F—C1F	−59.0 (7)	C2G—C3G—O3G—C1F	−142.5 (5)
O5G—C1G—C2G—O2G	177.9 (5)	O1G—C1G—O5G—C5G	62.6 (7)
O1G—C1G—C2G—O2G	53.1 (7)	C2G—C1G—O5G—C5G	−58.2 (7)
O5G—C1G—C2G—C3G	53.6 (7)	C6G—C5G—O5G—C1G	−179.8 (5)
O1G—C1G—C2G—C3G	−71.1 (6)	C4G—C5G—O5G—C1G	59.4 (7)

### Hydrogen-bond geometry (Å, °)

**Table:**

*D*—H···*A*	*D*—H	H···*A*	*D*···*A*	*D*—H···*A*
O3f—H3f1···O6g^i^	0.84	1.93	2.772 (7)	177
O4f—H4f1···O3f^ii^	0.84	2.04	2.880 (7)	175
O2g—H2g1···O2g^iii^	0.84	1.92	2.680 (7)	149
O4g—H4g1···O5f^iv^	0.84	2.08	2.827 (6)	149
O6g—H6g···O3f^v^	0.84	2.02	2.822 (7)	158

Symmetry codes: (i) −*x*+1/2, −*y*+1, *z*−1/2; (ii) *x*−1/2, −*y*+1/2, −*z*+1; (iii) *x*+1/2, −*y*+3/2, −*z*+1; (iv) *x*+1, *y*, *z*; (v) −*x*+3/2, −*y*+1, *z*+1/2.
